# Recycling honey bee drone brood for sustainable beekeeping

**DOI:** 10.1093/jee/toae303

**Published:** 2024-12-30

**Authors:** Ratko Pavlović, Karl Crailsheim, Miloš Petrović, Walter Goessler, Nenad M Zarić

**Affiliations:** Faculty of Chemistry, Department of Biochemistry, University of Belgrade, Belgrade, Serbia; Department of Biology, University of Graz, Graz, Austria; Faculty of Agriculture, University of Novi Sad, Novi Sad, Serbia; Institute of Chemistry, Analytical Chemistry for Health and Environment, University of Graz, Graz, Austria; Institute of Bioanalytics and Agro-Metabolomics, Department of Agrobiotechnology IFA-Tulln, University of Natural Resources and Life Sciences Vienna (BOKU), Tulln, Austria; Faculty of Biology, University of Belgrade, Belgrade, Serbia

**Keywords:** *Varroa*, mite, yellow mealworm, honey bee, nutrition

## Abstract

Pollination by insects is vital for global agriculture, with honey bees (*Apis mellifera* L.) being the most important pollinators. Honey bees are exposed to numerous stressors, including disease, pesticides, and inadequate nutrition, resulting in significant colony losses. This study investigates the use of drone brood to mitigate these problems. Drone brood, which is normally discarded during varroa mite (*Varroa destructor*, Anderson and Trueman) management, is rich in proteins, fats, and essential minerals. We compared drone brood with an already suggested pollen supplement (Tenebrio [*Tenebrio molitor* L.] flour). The results indicate that drone brood flour is a viable source of proteins, fats, and minerals and is potentially antimicrobial due to its high content of elements with known antimicrobial properties. It meets the nutritional needs of honey bees while mitigating the effects of varroa mites. The use of drone brood flour can provide high-quality beeswax, surplus of pollen, and improve bee health, which promotes sustainable beekeeping.

## Graphical Abstract

**Graphical Abstract:**
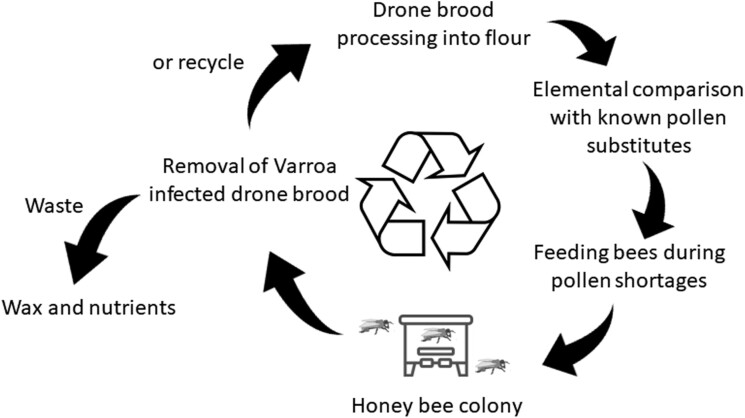

## Introduction

Insect pollination is crucial for global agriculture and human food security, with honey bees (*Apis mellifera* L. (Hymenoptera: Apidae)) being the most important pollinator species (Kleijn et al. 2015). Honey bees, like other pollinators, face various stressors, including a lack of foraging opportunities, constant exposure to diseases, pesticides, climate change, and inadequate beekeeping practices (Havard et al. 2020, Perkins et al. 2023, Shannon et al. 2023). These challenges put the global honey bee population under constant pressure, leading to periodic large colony losses (Bruckner et al. 2023, Gray et al. 2023, Aurell et al. 2024a). Malnutrition can directly affect the health of bees or indirectly weaken their immune response (Alaux et al. 2010, Goulson et al. 2015).

In nature, the bees’ diet consists mainly of nectar, pollen, honeydew, and water. This combination provides a complete source of macro- and micronutrients (Brodschneider and Crailsheim 2010). Nectar and honeydew meet their carbohydrate requirements, while pollen is an essential source of proteins, fats, vitamins, and minerals (Herbert and Hill 2015). A deficiency in any of these key elements often leads to a decline in the number of bees in a colony, a shortening of the lifespan of worker bees, lower tolerance to diseases and various pesticides, and ultimately the death of the colony (Brodschneider and Crailsheim 2010, Tsuruda et al. 2021). For reasons of cost efficiency, beekeepers sometimes keep hundreds of hives in a single location, but in this situation, the bees often rely on the beekeeper to provide them with carbohydrates and proteins (Sammataro and Weiss 2013). Bees can be fed different protein sources such as soy, yeast, and whey to replace pollen and different carbohydrate sources, such as sucrose and invert sugar syrup to replace nectar. Although various diet combinations have been developed and described, there is no true substitute that can fully replace the nutrients provided by pollen (Brodschneider and Crailsheim 2010, Noordyke and Ellis 2021). Pollen nutrition is a crucial factor for honey bee health (Di Pasquale et al. 2016, Barroso-Arévalo et al. 2019). Therefore, the ideal pollen supplement must have properties that attract honey bees, provide adequate nutrition, and at the same time be reasonably affordable (Ricigliano et al. 2021). Most of the commonly used recipes not only have a high carbon footprint (Vauterin et al. 2021) but are also based on a combination of soybeans, milk powder, linseed oil, and brewer’s yeast (Brodschneider and Crailsheim 2010). The inclusion of components that correlate more with the ecology and physiology of bees is necessary.

Edible insects have emerged as a promising alternative source of protein due to their low environmental footprint, high feed conversion rate, low land use, and ability to process low-value organic waste (Tiwasing and Pate 2024). They are widely used as a protein component in complete feeds in aquaculture, poultry production, and the pet industry (van Huis 2020, Nasir et al. 2024). Their use as honey bee feed was just recently suggested (Pavlović et al. 2023). For this purpose, yellow mealworm (*Tenebrio molitor* L. [Coleoptera: Tenebrionidae]) was used. The idea of including edible insects in the diet of honey bees is based on their known cannibalistic behavior (Schmickl and Crailsheim 2001). When pollen stops coming into the hive, the bees start cannibalizing larvae younger than 3 days old after 5 days (Schmickl and Crailsheim 2001). This helps the bees to make optimum use of the available nutrients in the colony. Cannibalism in honey bees is not limited to this situation. When a queen mates with closely related drones, the resulting brood has a lower survival rate because the eggs develop into diploid drones instead of workers. Worker bees soon eat all these larvae (Woyke 1963). Furthermore, if a queen lays more than one egg in a cell, the bees gradually eat the excess eggs and the larvae that hatch from them. No more than one larva remains in a cell for more than 4 days after hatching (Lehnert 1965). Eggs laid at the edges of the brood, where they are not well covered by the workers, are often eaten as well (Fukuda and Sakagami 1968).

In addition to malnutrition, another major threat to the global honey bee population is the parasitic mite Varroa (*Varroa destructor*, Anderson and Trueman [Mesostigmata: Varroidae]), which is considered by many scientists to be the main suspect for colony losses. The pathogen has 2 life stages, one on the adult bees and a reproductive stage in sealed drone and/or worker brood (Rosenkranz et al. 2010). During this reproductive stage, Varroa females prefer drone larvae since its reproductive stage is longer compared to worker larvae (Calderone and Kuenen 2001). This increases the mites’ chances of successful reproduction (Fuchs 1992, Odemer et al. 2022).

Safe, reliable, and simple control methods that can completely eradicate this pest from hives have not yet been developed. The commonly used synthetic acaricides, such as fluvalinate, amitraz, and coumaphos fail because the mite populations can quickly develop resistance (Rodríguez-Dehaibes et al. 2005, Mitton et al. 2018). In addition to the direct damage caused by Varroa feeding on honey bees, colonies often collapse because the mite is a vector for the transmission of deadly viruses (Martin et al. 2012). Hence, a rapid control can help to reduce damage caused by Varroa (Aurell et al. 2024b). However, the accumulation of acaricides used to control Varroa together with antimicrobial drugs and fungicides leads to a synergistic effect that results in the pesticides becoming more toxic to honey bees (Johnson et al. 2013).

Another approach to Varroa mite control is integrated pest management (IPM), which uses multiple non-chemical tools that have been shown to control the mite population (Jack and Ellis 2021, Vilarem et al. 2021). The main benefit of IPM strategies is the direct reduction in the use of pesticides responsible for the contamination of hive products, such as honey, pollen, and wax. IPM relies, among other things, on the removal of capped drone brood, which has often been shown to be effective (Wantuch and Tarpy 2009). Varroa mites are 5 to 12 times more abundant on drone brood compared to worker brood (Calderone and Kuenen 2001). They prefer to reproduce in drone broods and are attracted to it. Shortly before the bees cap the brood, the mites enter and begin to feed and reproduce. As the development of the drones takes longer than that of the worker bees, the mites produce more offspring in the drone brood than in the worker brood. Thus, removing capped drone brood from an infected colony removes a disproportionate number of mites without affecting the size of worker populations or honey production (Calderone 2005, Jack and Ellis 2021). However, this method has the major disadvantage that large amounts of nutrients are also wasted in this way. Nevertheless, beekeepers remove large quantities of drone brood every year.

The aim of this study is to find a sustainable way to combat 2 major threats to honey bee colonies, namely poor nutrition and the Varroa mite, and to explore a method of utilizing drone brood to combat these 2 threats to honey bee colony survival simultaneously.

## Materials and Methods

### Drone Brood Rearing and Flour Preparation

Foundationless wooden frames were inserted at 5 honey bee (*Apis mellifera* L.) colonies, at the same apiary located in Vršac, Serbia (45°06ʹ35.3″N 21°18ʹ27.0″E), beside worker brood frames as suggested by Jensen et al. (2019). The apiary consisting of 15 colonies was surrounded by a variety of different plant species. After 25 days a total of 1.50 kg of capped drone brood was collected from 5 colonies and combined. Larvae and pupae were checked for the presence of varroa mite. To remove the wax, the drone brood was boiled in 4.5 L of water. As soon as the water started boiling, the mixture of water, wax, drone larvae, and pupae was strained. The procedure was repeated once more with 3 L of water. Wax was removed. Cooked larvae and pupae were dried at 80°C for 3 h using an electronic food dryer designed for household use and afterward were ground into flour.

### *Varroa destructor* Assessment

After separating drone larvae and pupae from the wax, it can be seen that they are enclosed in a cocoon. The cocoons were removed from a total of 100 randomly selected larvae and pupae to see if they were infected with *Varroa destructor* (from now on Varroa).

### Yellow Mealworm (*Tenebrio molitor* L.) Rearing and Flour Preparation

Larvae of yellow mealworm (*Tenebrio molitor* L.), which were used as a control (from now on Tenebrio) were reared at the Laboratory of Entomology, Faculty of Agriculture, University of Novi Sad adopted from Jajić et al. (2022). The rearing cycle started with 10-day-old adults, who were put on a food substrate consisting of a mixture of wheat bran (95%) with brewer’s yeast (5%). The mixture was evenly spread in 5 standard insect-rearing plastic trays whose dimensions were 10 × 50 × 60 cm. The trays were put in a climate chamber at 26°C and 60% relative humidity. The adults were left 7 days on a selected mixture to copulate and lay eggs. After 7 days, adults were removed from the trays, and the trays were returned to the climate chamber for eggs to complete the embryogenesis and neonatal larvae to hatch. The larvae were provided with fresh apple slices 3 times a week as a source of moisture. On days 30 and 60 after removing the adults, the food substrate was sieved and removed, and the fresh quantity was added. The rearing cycle lasted 90 days (Knežić et al. 2024).

The 90-day-old larvae from 5 trays were combined and sieved from food substrate and put in a clean tray for the starvation procedure, which lasted for 24 h. The purpose was to eliminate the remaining feces from the digestive tract. The remaining feces was sieved and, the larvae were firstly washed under running water and then put in an industrial fryer AEF-360, which was set up to boil water and maintain the water temperature at 100°C. The larvae were blanched in water for 180 s and after that were put on drying trays for 30 min. The cooked larvae were oven dried and ground into flour.

### Element Concentrations

#### Chemicals and Standards

A purification system (Milli-Q, Merck Millipore, Darmstadt, Germany) was used to provide purified water (18.2 MΩ cm). Nitric acid (HNO_3_) Rotipuran p. a. ≥ 65% (Carl Roth, Karlsruhe, Germany) was subboiled with an MLS duoPUR (MLS, Leutkirch, Germany) prior to its use for the preparation of samples. For internal standards and preparation of calibration standards, ICP Single-Element Standards Certipur (Merck Millipore, Darmstadt, Germany) and Single Element Standards for ICP (Carl Roth, Karlsruhe, Germany) were used. Fifteen and 50 ml Cellstar polypropylene tubes (Greiner Bio-One International GmbH, Kremsmünster, Austria) were used.

#### Sample Preparation

From homogenized samples of drone brood flour and Tenebrio flour, 5 replicates each were prepared according to Zarić et al. (2022a). In short, 100 mg of freeze-dried and homogenized Tenebrio and drone brood flour samples were digested in an ultraCLAVE IV microwave digestion system (MLS GmbH, Leutkirch, Germany) using 5 ml of concentrated HNO_3_. Each digestion was accompanied by 3 digestion blanks (5 ml conc. HNO_3_) and 3 reference materials BOVM-1 “Bovine muscle powder” (NRC, Canada). After digestion samples were left to cool, afterward, they were transferred to 50 ml Cellstar tubes and diluted with ultrapure water to a final volume of 50 ml (10% (v/v) nitric acid). The same procedure was used for all samples.

#### Determination of Element Concentrations

Element concentrations were determined as described by Zarić et al. (2022b) using inductively coupled plasma mass spectrometry—ICPMS (Agilent ICPMS 7700x, Waldbronn, Germany). Instrument performance is reported in Supplementary Tables S1 and S2. External calibration curves with 6 points and 4 concentration ranges were used (Supplementary material, Text S1). The calibration curve was made in 10% HNO_3_ to match the sample matrix.

#### Quality Control

Quality control was achieved as already described by Zarić et al. (2022b), through the continuous addition of internal standards (Be, Ge, In, and Lu (200 μg L^−1^ in 1% v/v HNO_3_)), and the analyses of drift standards (after every 10 samples). Accuracy was evaluated by subjecting BOVM-1: Bovine Muscle Certified Reference Material for Trace Metals and other Constituents (NRC, Canada) through the same digestion process as the samples (Supplementary Table S4). In addition, accuracy was also evaluated using SRM 1643f Trace elements in natural water (National Institute of Standards & Technology, Gaithersburg, USA) (Supplementary Table S3).

### Determination of Total Fat

An automated Soxtherm fat extraction system (Gerhardt, Germany) was used, following Thiex et al. (2003). Two grams of sample (Tenebrio or drone flour) in triplicate was extracted using 120 ml petroleum ether. After extraction, the extract was dried in the oven until constant mass. Prior to gravimetric fat determination beakers were cooled down, weighed, and the percent total fat was calculated using the formula:


$$\begin{array}{l} \;\%\;crude\;fat\;=\;\frac{\left(m2-m1\right)}{m0}\;\times \;100 \end{array}$$

where m0 = initial samples weight; m1 = weight of empty extraction beaker; m2 = weight of extraction beaker with fat after drying.

### Determination of Proteins

Kjeldahl method used for honey bee brood was adopted from Jensen et al. (2019). In short, 1 g of sample was digested in sulfuric acid with potassium sulphate, and copper sulphate as a catalyst. The distillate was diluted with water and then alkalized with sodium hydroxide solution. Ammonia distillation was performed, and distillate was collected in a boric acid solution. Titration was performed simultaneously. Samples were analyzed in triplicate.

The following formula was used for calculating total nitrogen:


$$\begin{array}{l} Ns=\frac{Vs-Vb\times 0.1\times 14.0007}{10\times Ws} \end{array}$$

where *N*_s_ is the total nitrogen in sample (%); *V*_s_ is the titration volume for sample (ml); *V*_b_ is the titration volume for blanks (ml); *W*_s_ is the sample weight (g). A conversion factor of 6.25 (0.16 g of nitrogen per gram of protein) was used to transform the nitrogen into protein content:


$$\begin{array}{l} Total\;protein,\;\%=Ns\;\times \;6.25 \end{array}$$

### Statistical Analyses

Element concentrations were compared using IBM SPSS 29. Input data were elements concentration in 5 replicate samples of drone flour and 5 replicate samples of Tenebrio flour. To assess normality Kolmogorov–Smirnov test was used. Most of the data were normally distributed. Hence, an independent samples *t*-test was used. A correction for multiple comparisons was done by calculating the Bonferroni-corrected *P*-value. For the data points that were below the detection limit, half of the detection limit value was used.

## Results

**Fig. 1.**
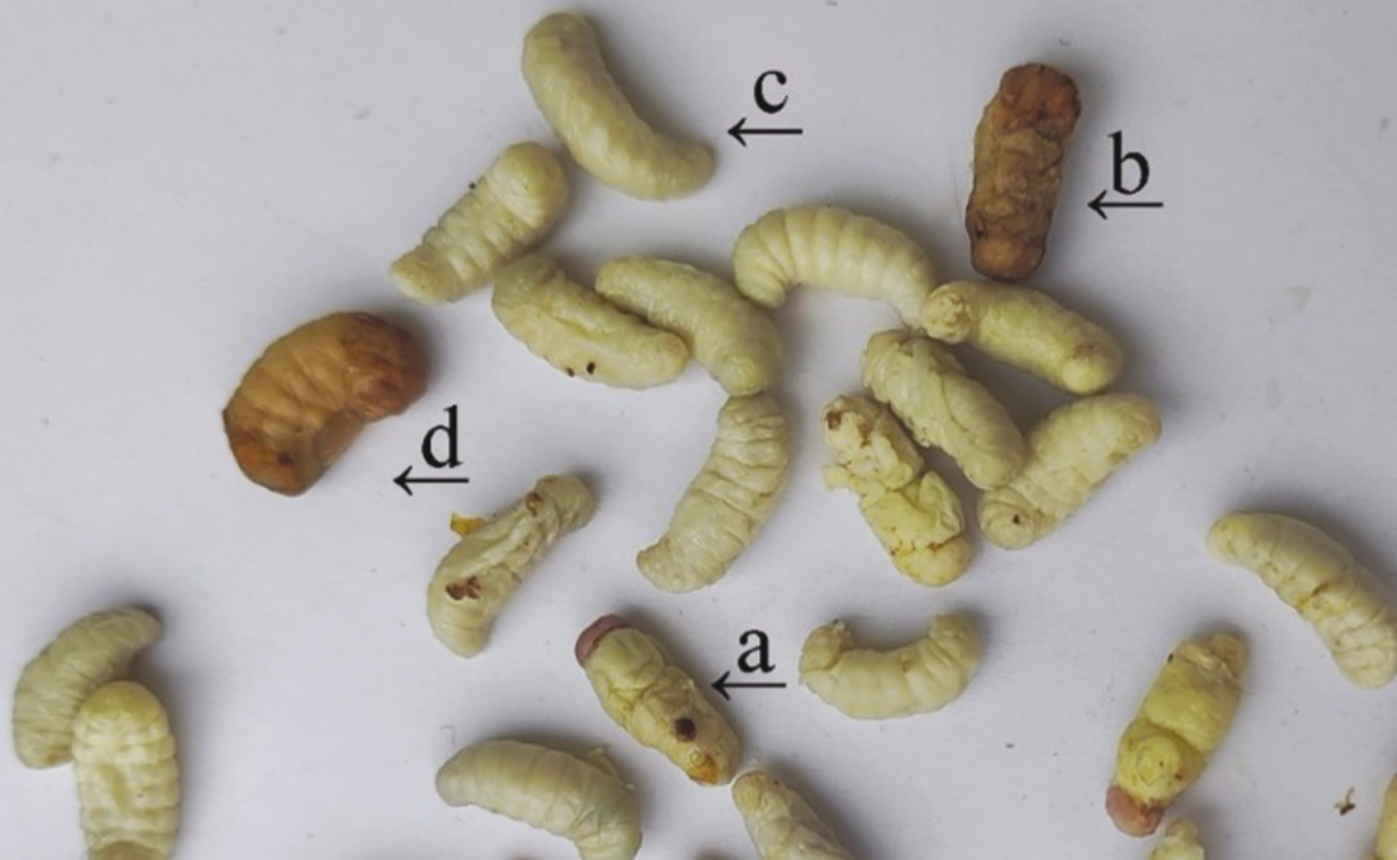
Drone brood (*Apis mellifera*) after cooking and straining: (a) Drone pupa with varroa mite after cocoon removal, (b) Drone pupa enveloped in cocoon, (c) Drone larva after cocoon removal, (d) Drone larva enveloped in cocoon.

After the assessment of the presence of Varroa, a total of 4 mites were found (Fig. 1).

In our pilot case, we obtained 1,130 g of cooked larvae and pupae from about 1,500 g of drone brood. This yielded 300 g of dried drone larvae and pupae (about 20% of the original mass of capped drone brood removed from the hive) and 130 g of beeswax.

All elements analyzed were detected in drone flour. In Tenebrio flour (control) V and Ag were below the detection limits, while Al was detected in only one sample. Element comparison between drone and Tenebrio flour showed significant differences for most elements according to an independent *t*-test (Table 1).

**Table 1.** Results of the independent *t* test on differences in elemental concentrations between Tenebrio and drone (*Apis mellifera*) flour.

Element	Independent *t* test
Li^*^	*t*(8) = 23.071, *P* < 0.0001
B	*t*(8) = 2.801, *P* = 0.024
Na^*^	*t*(8) = −85.809, *P* < 0.0001
Mg^*^	*t*(8) = −116.085, *P* < 0.0001
Al^*^	*t*(8) = 9.531, *P* = 0.001
P^*^	*t*(8) = −68.171, *P* < 0.0001
S^*^	*t*(8) = −10.722, *P* < 0.0001
K^*^	*t*(8) = −34.283, *P* < 0.0001
Ca^*^	*t*(8) = 13.870, *P* < 0.0001
V	*t*(8) = 1.775, *P* = 0.114
Cr^*^	*t*(8) = 6.699, *P* = 0.0002
Mn^*^	*t*(8) = −53.056, *P* < 0.0001
Fe	*t*(8) = −3.881, *P* = 0.005
Co^*^	*t*(8) = 21.885, *P* < 0.0001
Ni^*^	*t*(8) = −19.040, *P* < 0.0001
Cu^*^	*t*(8) = −43.645, *P* < 0.0001
Zn^*^	*t*(8) = −44.875, *P* < 0.0001
As^*^	*t*(8) = −19.748, *P* < 0.0001
Se^*^	*t*(8) = −75.394, *P* < 0.0001
Rb^*^	*t*(8) = 19.499, *P* < 0.0001
Sr^*^	*t*(8) = 8.022, *P* < 0.0001
Mo^*^	*t*(8) = −188.368, *P* < 0.0001
Ag^*^	*t*(8) = 51.216, *P* < 0.0001
Cd^*^	*t*(8) = −40.442, *P* < 0.0001
Sn	*t*(8) = 3.148, *P* = 0.014
Sb	*t*(8) = 4.352, *P* = 0.002
Ba	*t*(8) = 0.412, *P* = 0.691
Pb^*^	*t*(8) = 38.468, *P* < 0.0001

^*^Statistically significant differences in accordance with Bonferroni-corrected *P*-value (*P* < 0.0018).

**Fig. 2.**
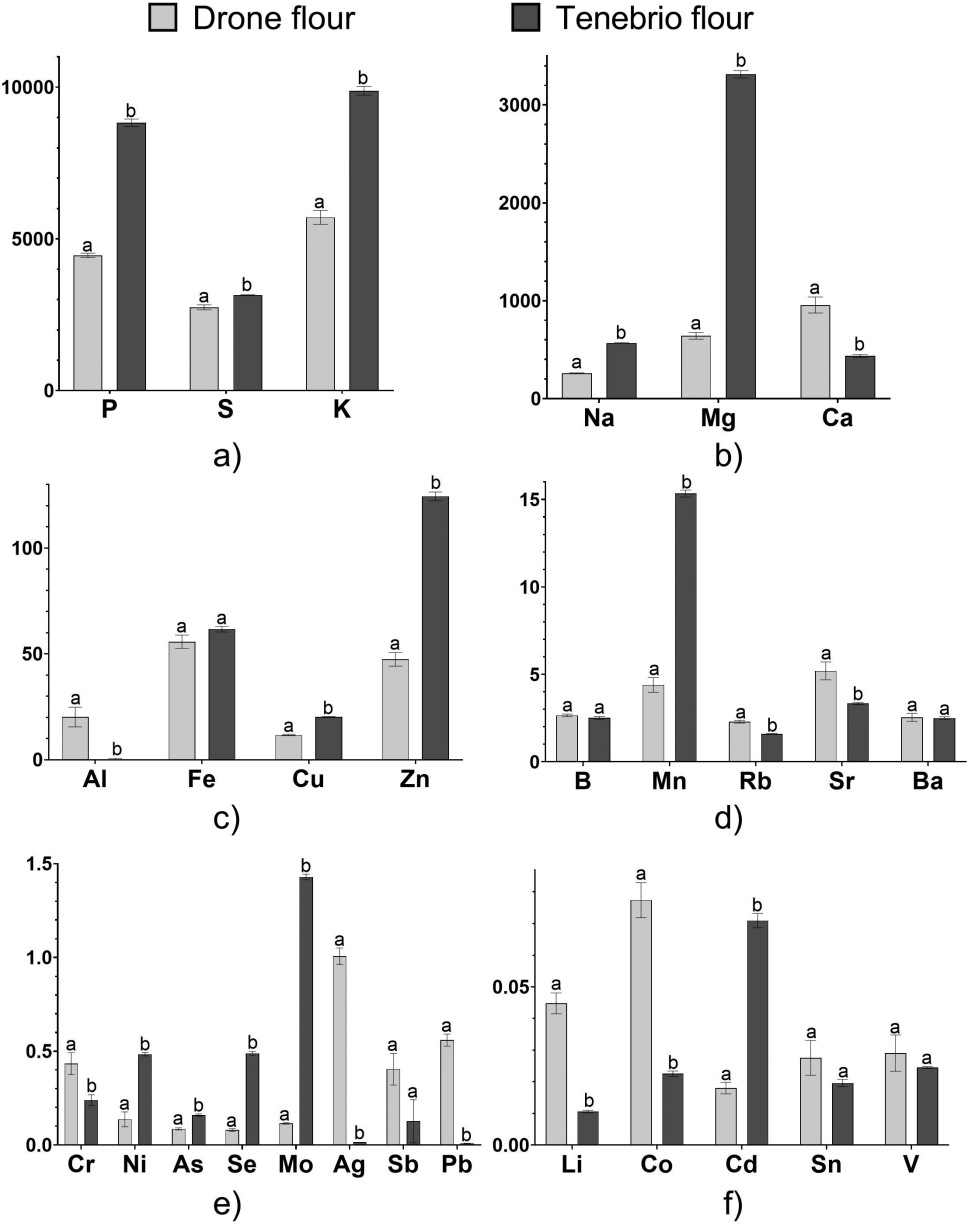
(a)–(f) Element concentrations (mg kg^−1^ dry weight) in Drone (*Apis mellifera*) and Tenebrio flour; different lower-case letters (a and b) represent statistically significant differences in element concentrations (per element analyzed) between Drone and Tenebrio flour.

Drone flour contained statistically higher concentrations of Ca, Al, Rb, Sr, Cr, Ag, Sb, Pb, Li, and Co. Tenebrio flour had higher concentrations of P, S, K, Na, Mg, Cu, Zn, Mn, Ni, As, Se, Mo, and Cd (Table 1). There was no difference for B, V, Fe, Sn, Sb, and Ba (*P* > 0.0018) (Fig. 2). The most abundant elements were S, P, K, Mg, Ca, and Na, which account for 98.96% of the total amount of elements analyzed in drone flour and 99.11% of the total amount of elements analyzed in Tenebrio flour.

The analyses showed that the dried drone brood contained an average of 35.5% proteins and 40.6% fat. Dried Tenebrio flour contained an average of 46.4% protein and 21.7% fat.

## Discussion

If the total biomass of drone brood collected per colony in a season is 1,064 g (Lecocq et al. 2018), we can estimate, based on our preliminary data, that at least 200 g of drone flour could be produced from each hive per season. The protein, lipid, and micronutrient content in this drone flour could be used to supplement a hive during periods of pollen scarcity.

Use of drone brood as a pollen supplement is based on honey bee cannibalistic behavior. The potential downside is the spread diseases, especially deformed wing virus through worker bees consuming pupae (Posada-Florez et al. 2021). However, our method most likely mitigates this possibility, since separating the wax from the pupae drone brood is done by cooking it at 100°C twice and afterward drying it at a temperature of 80°C.

Our observations, based on only one colony of ~1,200 g of bees, were that they consumed 1,435 g of commercial sugar patty containing 4% of drone brood flour. This shows that drone flour is well accepted by honey bees because the consumption is only around 22% lower compared to the same patty mixed with 4% pollen (1,857 g). However, further studies on consumption are needed to verify these findings.

Pollen usually contains between 2% and 60% protein and 2% and 20% lipids (Roulston and Cane 2000). Bees’ usual diet comprised around 2:1 ratio of protein to lipids (Wright et al. 2018). However, bees can regulate their intake of protein and lipids (Stabler et al. 2021). In addition, when used as feed, the amount of protein and lipids can be easily adjusted to the desired content by mixing drone brood flour with sugar patty (Zhao et al. 2016). Not all lipids are useful to bees to the same extent. Hence, further study is needed to determine the lipid profile of drone brood in comparison to that of pollen. Besides proteins and lipids, drone brood flour also contains minerals and micronutrients that are important for the reproduction and development of adult bees and larvae (Ilijević et al. 2021).

A study done by Filipiak et al. (2017) showed that elements that limit honey bee development due to their scarcity of pollen are Na, S, K, P, Cu, and possibly Zn. From our unpublished data on element concentrations in pollen (Supplementary Table S5), it can be observed that all these elements had at least 2× higher concentrations in drone and Tenebrio flour compared to pollen. However, not only elemental concentrations but their stoichiometry plays a role in bee nutrition (Filipiak 2018). A low ratio of K:Na was proven to be significant to bees (Filipiak et al. 2023). This ratio in drone flour is two times lower compared to pollen (22 and 48, respectively). This suggests that the use of drone flour as a pollen supplement could improve honey bee development.

Although they are very important, the role of micronutrients in honey bee health is not yet fully understood (Khan et al. 2021). In our previous study (Pavlović et al. 2024), we analyzed the effects of micronutrients on the onset of chalkbrood disease in honey bees. We found that chalkbrood mummies and larvae from infected hives were significantly deficient in elements (Al, Zn, Mn, and Cr), which have known antifungal and antimicrobial properties (Pavlović et al. 2024). Drone flour contains higher concentrations of many of these elements, whose deficiency might contribute to the onset of chalkbrood disease (Fig. 2). Comparing the elemental composition of drone and Tenebrio flour with that of pollen, it is interesting to note that Ba, Zn, and Ag have higher concentrations in the flours, with Ag being almost absent in pollen (Temizer et al. 2018, Aldgini et al. 2019). This suggests that drone brood flour, rich in these elements, could play an additional role in the hive as a potential source of antimicrobial elements, helping in the prevention and control of disease. This is supported by the findings that the addition of drone brood flour to pork patties significantly reduces total microbial count (Choi et al. 2024).

Based on the elemental composition and the natural cannibalistic behavior of honey bees, it can be concluded that drone brood flour can be a suitable supplement for pollen, possibly even better than Tenebrio flour (used as control), which has already shown good results (Pavlović et al. 2023). We have already observed that caged bees can survive for at least 28 days on a diet consisting solely of a mixture of 12.5% Tenebrio flour and commercial sugar patties. These bees maintained a higher body weight and the highest abdomen weight compared to other tested pollen substitutes, demonstrating the best overall performance among the tested patties (Pavlović et al. 2023). To determine how drone flour compares to Tenebrio flour, further study is needed.

The second benefit of removing drone brood is to combat Varroa mite infestation. Although this practice has a negative effect on honey production, providing colonies with drone combs may still be beneficial, as the practice of removing drone brood to kill mites could balance the negative effects of drone combs on honey yields (Seeley 2002). Although pupae on which Varroa parasites live have a lower mass, the impact of Varroa on their nutritional value, mostly mineral content, is insignificant (Jensen et al. 2019). In most cases, after removing the drone brood, beekeepers are left with beeswax and a waste product: drone larvae and pupae.

Usually, most of the drone brood is wasted after collection. Meanwhile, many beekeepers need to provide their colonies with food supplements, sometimes year-round. Even when not strictly necessary, increasing pollen collection from hives without compromising bee nutrition can boost beekeepers’ profits, as surplus pollen is becoming increasingly popular as a health product. By using drone flour, beekeepers can reduce their reliance on expensive commercial supplements, which are made from high-carbon footprint ingredients. In addition, since the collection and use of drone flour occur within the same apiary, there are no transportation costs. The process for producing this feed is technically simple and can be done in almost any apiary with access to simple household items that include pots, strainers, food dryer, and grinder.

Drone brood removal offers proven benefits, such as reducing varroa mite populations, minimizing pesticide use, and obtaining high-quality wax (Calderone 2005, Rosenkranz et al. 2010). In addition, it can serve a new important purpose for beekeepers: as a pollen supplement. We suggest using this byproduct as bee feed in times of pollen scarcity. Bee brood is an excellent source of many valuable nutrients and is high in proteins and fats. This study also highlights its high mineral content. In addition, it is rich in elements with antimicrobial and antifungal properties that could raise honey bees’ resilience to diseases. The separation of larvae and pupae from the wax is a simple process that can be carried out with standard household equipment and is therefore accessible to all beekeepers.

## Supplementary data

Supplementary data are available at *Journal of Economic Entomology* online.

toae303_suppl_Supplementary_Material
