# Supplementary material for: Recycling honey bee drone brood for sustainable beekeeping
Source: J Econ Entomol. 2024 Dec 30;118(1):37–44. doi: 10.1093/jee/toae303 (PMC11818385; doi:10.1093/jee/toae303)
Supplement: toae303_suppl_Supplementary_Material [file toae303_suppl_supplementary_material.docx]

Text S1:

Calibration curve ranges:

0.0100–5.00 μg L^−1^: Li, V, Cr, Co, Ni, As, Se, Mo, Ag, Cd, Sn, Sb, Cs, Tl, Pb and U

0.1–50 μg L^−1^: B, Ba, Cu, Rb, and Sr

1.00–500 μg L^−1^: Al, Mn, Fe, Zn

100–50,000 μg L^−1^: Na, K, Ca, Mg, P and S

Table S1. Performance of the ICPMS

| **Parameter** | **No-gas mode** | **Collision mode** | **Reaction mode** |
| --- | --- | --- | --- |
| Cell gas | - | He | H_2_ |
| ^7^Li [CPS per µg L^-1­^] | 2.8*10^3^ | - | - |
| ^59^Co [CPS per µg L^-1^] | - | 2.6*10^3^ | 4.5*10^2^ |
| ^89^Y [CPS per µg L^-1^] | 17*10^3^ | 3.3*10^3^ | 7.2*10^3^ |
| ^205^Tl [CPS per µg L^-1^] | 7.8*10^3^ | 6.3*10^4^ | 7.2*10^4^ |
| average RSD [%] | 2.5 | 2.5 | 3.5 |
| ^140^Ce^16^O/^140^Ce [%] | 1.7 | 0.9 | 2.4 |
| ^140^Ce^2+^/^140^Ce^+^ [%] | 2.5 | 0.7 | 0.3 |

Table S2. Selected mass, tune mode, internal standard and detection limits (LoD)

| **Monitored isotope** | **Tune mode** | **Internal standard** | **Detection limit^*^**  **(µg L^-1^)** |
| --- | --- | --- | --- |
| ^7^Li | No-gas | ^9^Be | 0.0028 |
| ^11^B | No-gas | ^9^Be | 0.23 |
| ^23^Na | He | ^9^Be | 27 |
| ^24^Mg | He | ^9^Be | 11 |
| ^27^Al | No-gas | ^9^Be | 9.3 |
| ^31^P | He | ^9^Be | 27 |
| ^32^S | He | ^9^Be | 1641 |
| ^39^K | He | ^9^Be | 9.8 |
| ^43^Ca | He | ^9^Be | 154 |
| ^51^V | He | ^74^Ge | 0.02 |
| ^53^Cr | He | ^74^Ge | 0.08 |
| ^55^Mn | He | ^74^Ge | 0.09 |
| ^56^Fe | He | ^74^Ge | 2.7 |
| ^59^Co | He | ^74^Ge | 0.004 |
| ^60^Ni | He | ^74^Ge | 0.9 |
| ^65^Cu | He | ^74^Ge | 0.62 |
| ^66^Zn | He | ^74^Ge | 9.3 |
| ^75^As | He | ^74^Ge | 0.006 |
| ^78^Se | H_2_ | ^74^Ge | 0.01 |
| ^85^Rb | He | ^74^Ge | 0.023 |
| ^88^Sr | He | ^74^Ge | 0.1 |
| ^98^Mo | No-gas | ^74^Ge | 0.02 |
| ^107^Ag | No-gas | ^115^In | 0.003 |
| ^111^Cd | No-gas | ^115^In | 0.009 |
| ^118^Sn | No-gas | ^115^In | 0.05 |
| ^121^Sb | No-gas | ^115^In | 0.02 |
| ^133^Cs | No-gas | ^115^In | 0.002 |
| ^137^Ba | No-gas | ^115^In | 0.17 |
| ^205^Tl | No-gas | ^175^Lu | 0.0012 |
| ^208^Pb | No-gas | ^175^Lu | 0.03 |
| ^238^U | No-gas | ^175^Lu | 0.0003 |

^*^LoD = mean_blanks_ + 3*σ_blanks_

Table S3. Certified and determined values for elements in NIST SRM 1643f Trace Elements in Natural Water

| **Element** | **Cert. mass conc. [µg kg^-1^]** | | | **Analyzed mass conc. [µg kg^-1­^] (n=3)** | | |
| --- | --- | --- | --- | --- | --- | --- |
| Li | 16.42 | ± | 0.35 | 15.93 | ± | 0.16 |
| B | 150.8 | ± | 6.6 | 140.5 | ± | 1.4 |
| Na | 18640 | ± | 240 | 23572 | ± | 872 |
| Mg | 7380 | ± | 58 | 7242 | ± | 333 |
| Al | 132.5 | ± | 1.2 | 139.8 | ± | 2.2 |
| K | 1913.3 | ± | 9.0 | 2041 | ± | 51 |
| Ca | 29140 | ± | 320 | 28440 | ± | 170 |
| V | 35.71 | ± | 0.27 | 32.32 | ± | 0.71 |
| Cr | 18.32 | ± | 0.10 | 17.12 | ± | 0.38 |
| Mn | 36.77 | ± | 0.58 | 36.9 | ± | 1.2 |
| Fe | 92.51 | ± | 0.77 | 90.7 | ± | 2.2 |
| Co | 25.05 | ± | 0.17 | 23.30 | ± | 0.42 |
| Ni | 59.2 | ± | 1.4 | 52.68 | ± | 0.95 |
| Cu | 21.44 | ± | 0.70 | 21.39 | ± | 0.53 |
| Zn | 73.7 | ± | 1.7 | 331.3 | ± | 6.3 |
| As | 56.85 | ± | 0.37 | 55.9 | ± | 1.6 |
| Se | 11.583 | ± | 0.078 | 10.82 | ± | 0.41 |
| Rb | 12.51 | ± | 0.12 | 11.94 | ± | 0.31 |
| Sr | 311 | ± | 18 | 183.2 | ± | 6.2 |
| Mo | 114.2 | ± | 1.7 | 109.26 | ± | 0.76 |
| Ag | 0.9606 | ± | 0.0053 | 1.320 | ± | 0.026 |
| Cd | 5.83 | ± | 0.13 | 7.17 | ± | 0.12 |
| Sb | 54.90 | ± | 0.39 | 54.36 | ± | 0.38 |
| Ba | 513.1 | ± | 7.3 | 467.2 | ± | 4.7 |
| Tl | 6.823 | ± | 0.034 | 6.715 | ± | 0.060 |
| Pb | 18.303 | ± | 0.081 | 19.52 | ± | 0.23 |

Table S4 Certified and determined values for elements in CRM BOVN-1 Bovine Muscle Powder (*information values)

| **Element** | **Cert. mass conc. [mg kg^-1^]** | | | **Analyzed mass conc. [mg kg^-1­^] (n=6)** | | |
| --- | --- | --- | --- | --- | --- | --- |
| B | 600 | ± | 400 | 236 | ± | 37 |
| Na | 2100 | ± | 100 | 1779 | ± | 14 |
| Mg | 960 | ± | 95 | 875.1 | ± | 76 |
| Al* | 1.7 | ± | / | 0.82 | ± | 0.38 |
| P | 8360 | ± | 450 | 7055 | ± | 51 |
| S* | 8000 | ± | / | 6787 | ± | 63 |
| K | 15200 | ± | 400 | 13691 | ± | 110 |
| Ca | 145 | ± | 20 | 130.2 | ± | 7.8 |
| V* | 0.005 | ± | / | 0.0030 | ± | 0.0013 |
| Cr* | 0.071 | ± | / | 0.053 | ± | 0.036 |
| Mn | 0.37 | ± | 0.09 | 0.308 | ± | 0.011 |
| Fe | 71.2 | ± | 9.2 | 63.2 | ± | 1.3 |
| Co | 0.007 | ± | 0.003 | 0.00552 | ± | 0.00022 |
| Ni* | 0.05 | ± | / | 0 | ± | / |
| Cu | 2.84 | ± | 0.45 | 2.412 | ± | 0.021 |
| Zn | 142 | ± | 14 | 130.4 | ± | 1.3 |
| As | 0.009 | ± | 0.003 | 0.0085 | ± | 0.0013 |
| Se | 0.076 | ± | 0.010 | 0.0625 | ± | 0.0040 |
| Rb | 28.7 | ± | 3.5 | 24.23 | ± | 0.35 |
| Sr | 0.052 | ± | 0.015 | 0.0517 | ± | 0.0052 |
| Mo | 0.08 | ± | 0.06 | 0.0618 | ± | 0.0017 |
| Cd | 0.013 | ± | 0.011 | 0.01045 | ± | 0.00098 |
| Sb* | 0.01 | ± | / | 0.0038 | ± | 0.0038 |
| Cs* | 0.05 | ± | / | 0.03070 | ± | 0.00042 |
| Ba* | 0.05 | ± | / | 0.0161 | ± | 0.0057 |
| Pb | 0.38 | ± | 0.024 | 0.39 | ± | 0.13 |

Table S5. Unpublished results on elements concentrations (mg kg^-1^ ± standard deviation (SD)) in pollen

| Element | Pollen | SD |
| --- | --- | --- |
| Na | 70.3 | 4.6 |
| P | 2398 | 44 |
| S | 1451 | 40 |
| K | 3426 | 31 |
| Cu | 7.679 | 0.051 |
| Zn | 22.0 | 3.6 |
